# Membrane-Bound Steel Factor Maintains a High Local Concentration for Mouse Primordial Germ Cell Motility, and Defines the Region of Their Migration

**DOI:** 10.1371/journal.pone.0025984

**Published:** 2011-10-06

**Authors:** Ying Gu, Christopher Runyan, Amanda Shoemaker, M. Azim Surani, Christopher Wylie

**Affiliations:** 1 Division of Developmental Biology, Cincinnati Children's Hospital Medical Center, Cincinnati, Ohio, United States of America; 2 Molecular and Developmental Biology Graduate Program, School of Medicine, University of Cincinnati, Cincinnati, Ohio, United States of America; 3 Division of Plastic Surgery, Cincinnati Children's Hospital Medical Center, Cincinnati, Ohio, United States of America; 4 Wellcome Trust/Cancer Research UK Gurdon Institute of Cancer and Developmental Biology, University of Cambridge, Cambridge, United Kingdom; Duke University Medical Center, United States of America

## Abstract

Steel factor, the protein product of the *Steel* locus in the mouse, is a multifunctional signal for the primordial germ cell population. We have shown previously that its expression accompanies the germ cells during migration to the gonads, forming a “travelling niche” that controls their survival, motility, and proliferation. Here we show that these functions are distributed between the alternatively spliced membrane-bound and soluble forms of Steel factor. The germ cells normally migrate as individuals from E7.5 to E11.5, when they aggregate together in the embryonic gonads. Movie analysis of *Steel-dickie* mutant embryos, which make only the soluble form, at E7.5, showed that the germ cells fail to migrate normally, and undergo “premature aggregation” in the base of the allantois. Survival and directionality of movement is not affected. Addition of excess soluble Steel factor to *Steel-dickie* embryos rescued germ cell motility, and addition of Steel factor to germ cells in vitro showed that a fourfold higher dose was required to increase motility, compared to survival. These data show that soluble Steel factor is sufficient for germ cell survival, and suggest that the membrane-bound form provides a higher local concentration of Steel factor that controls the balance between germ cell motility and aggregation. This hypothesis was tested by addition of excess soluble Steel factor to slice cultures of E11.5 embryos, when migration usually ceases, and the germ cells aggregate. This reversed the aggregation process, and caused increased motility of the germ cells. We conclude that the two forms of Steel factor control different aspects of germ cell behavior, and that membrane-bound Steel factor controls germ cell motility within a “motility niche” that moves through the embryo with the germ cells. Escape from this niche causes cessation of motility and death by apoptosis of the ectopic germ cells.

## Introduction

Primordial germ cells (PGCs) are the embryonic precursors of the gametes, and therefore play a central role in biology. In mice, PGCs are first specified in the extraembryonic allantois around E7.25, as a small group of cells that express characteristic markers such as alkaline phosphatase (AP) and Stella [1], [2]. They then migrate proximally into the posterior region of the embryo and become incorporated into the developing hind gut [3], [4]. Between E9.0 and E9.5, PGCs emigrate from the dorsal aspect of the hind gut and then migrate laterally through the dorsal body wall mesenchyme where they cease migration and aggregate together into clumps in the embryonic gonads [5]. During the four-day period of PGC migration, the embryo is undergoing rapid growth and organogenesis. The embryo grows more than six-fold in length during this period, and new tissues arise around PGCs as they migrate. PGC behavior, including proliferation, survival, motility and homing, are likely to be controlled by short-range signals in such a rapidly changing environment. We have shown previously that Steel factor provides such a short-range signal throughout the migratory period [4], [6],[7].

Mutations at the *Dominant white spotting* (*W*) and *Steel* (*Sl*) loci in the mouse cause failure of PGCs to colonize the embryonic gonads [8]–[13]. The W gene encodes c-kit, a cell-surface receptor tyrosine kinase expressed by PGCs throughout migration. The Steel gene encodes its protein ligand; Steel factor (also known as Stem Cell Factor, Kit-ligand, or Mast Cell Growth Factor). Previous studies from our laboratory and others revealed that Steel factor is an essential survival factor for PGCs *in vitro* [14]–[16]. More recently, we have shown that it also controls survival *in vitro*, and that its down-regulation in the midline at E10.5 causes death by apoptosis of ectopic PGCs still located there [7]. In addition to survival, Steel factor also controls motility and proliferation of the PGCs during migration [4], [7]. Steel factor is expressed by somatic cells immediately surrounding PGCs throughout their migration, from the time of PGC specification in the allantois, to the time they colonize the genital ridges, and is lost from these tissues after PGCs have moved on to the next location. Since PGC survival, proliferation and motility are all controlled by Steel factor signaling throughout migration [4], this suggests the existence of a “traveling niche” in which the Steel factor-expressing cells provide a continuous short-range signal to control different aspects of PGC behavior throughout their migration. This observation is supported by the fact that down-regulation of Steel factor in the midline at E10.5 causes the death by apoptosis of any ectopic germ cells still present in the midline [7]. However, the mechanism by which Steel factor plays such different roles in PGC behavior is still not understood.

The precursor RNA of Steel factor can be alternatively spliced to produce both soluble and transmembrane forms of the protein. The soluble form contains an extracellular domain with a proteolytic cleavage site which allows release of the extracellular region of the protein. The membrane-bound form lacks this domain, and therefore remains associated with the cell surface [17], [18]. Previous studies have shown that both forms of Steel factor are capable of activating c-kit, though the membrane-bound form tends to induce more persistent tyrosine kinase activation than the soluble form [19]. In some cell types, the two forms are thought to play different roles. For example, mast cells (which express c-kit) undergo initial adhesion to COS cells transfected with membrane-bound, but not soluble, Steel factor cDNA [17]. Similarly, membrane-bound Steel factor stimulates adhesion between hematopoietic stem cells and extracellular matrix [20]. It has also been reported that membrane-bound Steel factor, but not the soluble form, induces long-term proliferation of CD34^+^ cells [21]. In neural crest migration, the soluble form is essential for the initiation of melanocyte precursor dispersal onto the lateral pathway, while the membrane-bound form is required for their subsequent survival in the dermis [22]. It is not known why the membrane-bound and soluble forms have different properties in these model systems, or in most cases whether they do, in fact, play different roles in vivo. Steel factor has multiple functions in PGC behavior during migration, including survival, proliferation and motility, and it is not yet clear whether these different functions require specific forms of Steel factor.

The *Steel-dickie* (*Steel^d/d^*) mutation offers an opportunity to address this issue. This mutation carries a deletion of sequences that encode the intracellular and transmembrane domains of Steel factor, and therefore only produces a soluble truncated protein that lacks both the cytoplasmic and transmembrane domains [23]. *Steel^d/d^* mice are sterile, as they are in *Steel-null (Steel^-/-^*) mutants that lack the entire *Steel* gene. However, more PGCs are found in the gonad primordium in Steel^d/d^, suggesting some activity of the Steel factor signaling pathway remains in these mutants through its soluble form [11], [23]. In addition, *Steel^d/d^* mice are viable, which has suggested that Steel factor plays some role in PGC behavior that is not shared with hematopoietic cells. This has been a puzzling aspect of the *Steel^d/d^* mutation, since Steel factor is known to be a survival factor for both cell types.

In the present study, we first show by RT-PCR that both the membrane-bound and the soluble forms of Steel factor transcripts are expressed along the PGC migratory route. We then show that there is no significant change in PGC numbers between *Steel^d/d^* embryos and their wild type littermates at E7.5, suggesting that PGC survival requires only the soluble form of the protein at this stage. However, PGC numbers start to decrease in E8.0 *Steel^d/d^* embryos. Therefore some explanation must be found for the fact that they survive at E7.5, but die at later times. Time-lapse analysis of embryos at E7.5 shows that PGCs in *Steel^d/d^* embryos migrate at dramatically reduced rates, and instead aggregate into clumps in the allantois. As a result, they fail to migrate normally into the hind gut. This change in motility is the same as previously seen in *Steel-null* embryos, showing that PGC migration specifically requires the membrane-bound form of the protein. There are two possible reasons for this; either membrane-bound Steel factor confers a specific function on the cells adjacent to germ cells, perhaps altering adhesive properties for example, or it simply provides a higher local concentration of the protein immediately adjacent to the PGCs. To distinguish between these possibilities, we added increasing concentrations of recombinant soluble Steel factor to *Steel^d/d^* embryos cultured at E7.5, and showed that it rescued the defects of PGC motility in cultured *Steel^d/d^* embryos. Moreover, addition of soluble recombinant Steel factor into wild type embryo slices at E11.0 resulted in the reacquisition of motility in PGCs that had ceased migrating and aggregated in the genital ridges, and migration away from these sites of aggregation. Together, these data suggest that the primary role of membrane-bound Steel factor for PGC migration *in vivo* is to maintain an optimal local ligand concentration that promotes motility and inhibits aggregation in a restricted area of the embryo. It also shows that Steel factor controls different aspects of germ cell behavior at different concentrations. Low concentrations are sufficient to maintain survival, while higher concentrations provided by the membrane-bound form are required for motility.

## Results

### Both soluble and membrane-bound forms of Steel factor are present around PGCs throughout migration

**Figure 1:**
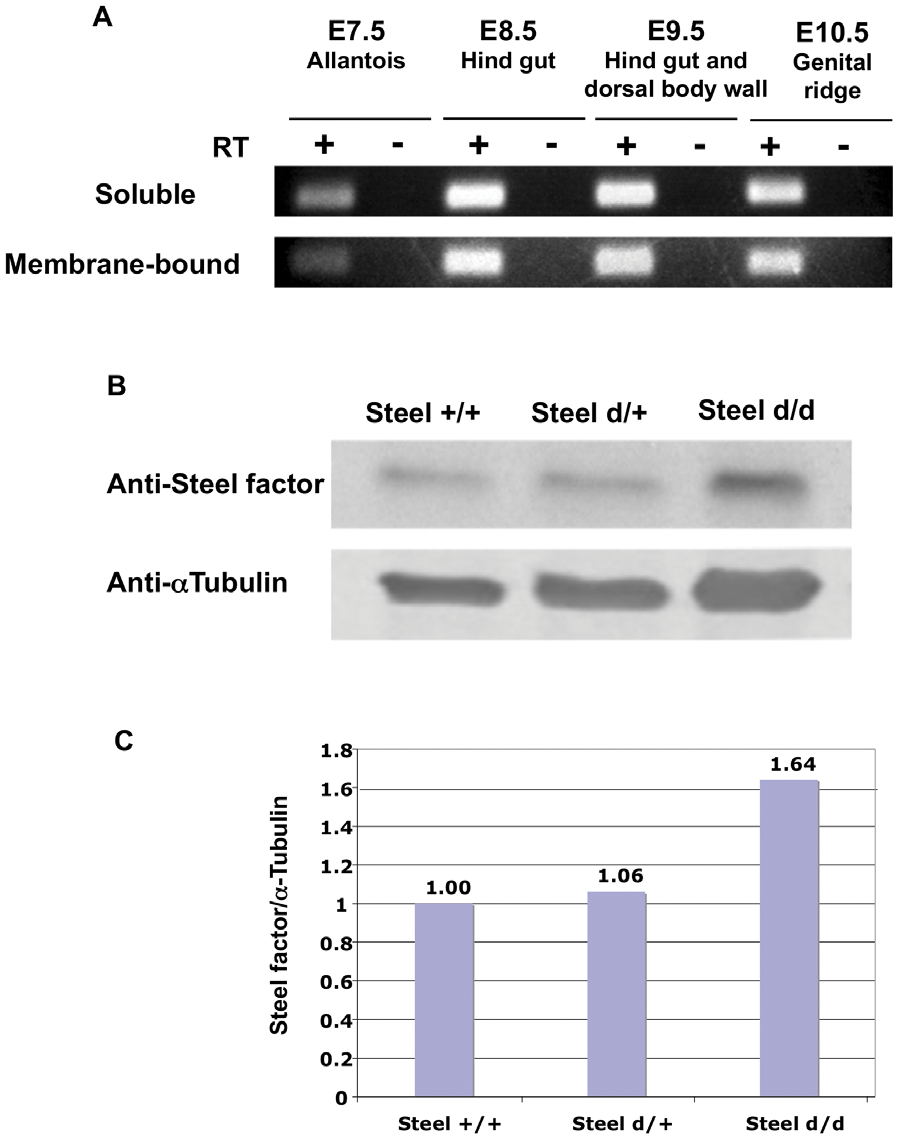
Expression of Steel factor in mouse embryos. (A) RT-PCR analysis of membrane-bound and soluble Steel factor. cDNA was prepared from dissected E7.5 allantois, E8.5 hind gut, E9.5 hind gut and dorsal body wall, and E10.5 genital ridge. (B) Expression levels of soluble Steel factor protein in embryos of different Steel-dickie genotypes measured by western blot. (C) Densitometric analysis of western blots in (B). α-tubulin antibody was used as a loading control.

Our previous study showed that both forms of Steel factor mRNA were present in the allantois at E7.5 by RT-PCR [4]. To investigate the expression pattern at later stages, we extracted RNA from tissues occupied by PGCs at all stages of migration and performed RT-PCR using primers that distinguish the two forms of Steel factor transcripts. Both transcripts were found in E7.5 allantoides, E8.5 hind gut endoderm, E9.5 hind guts and dorsal body walls, and E10.5 genital ridges (**Fig. 1A**), showing that both forms of Steel factor are present around PGCs throughout migration.

The *Steel-dickie* mutant makes only the soluble form. It has been shown previously by northern analysis that the *Steel-dickie* allele is expressed at the mRNA level at similar levels as the wild type [17], [23]; however, the protein levels were not previously assayed. We performed a western blot analysis of supernatant fractions of E12.5 embryos of different genotypes after triton extraction and high speed spin to remove the membranes. As shown in **Fig. 1B** and **1C**, there is no loss of Steel factor protein in *Steel^d/d^* embryos compared to their littermates, suggesting that there is no defect in mRNA translation in the *Steel^d/d^* embryos.

### Soluble Steel factor is sufficient to maintain PGC number at E7.5

**Figure 2:**
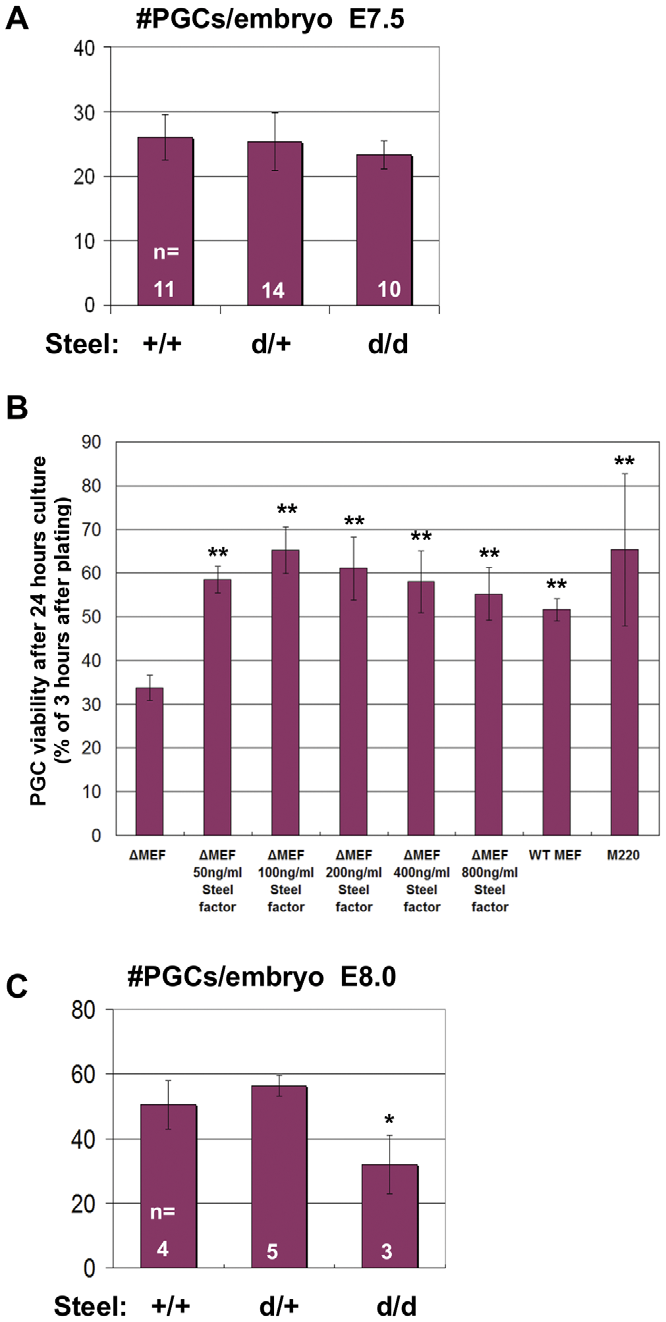
PGC number in *Steel^d/d^* embryos and *in vitro* culture. (A) There was no significant change in PGC numbers at E7.5 in Steel^d/d^ embryos compared to their littermates. “n” indicates the number of embryos used for quantitation. (B) PGC number after 24 hours in vitro culture in medium with or without soluble recombinant Steel factor on different feeder layer cells. Y axis represents the ratio of PGC number 24 hours after plating versus 3 hours after plating. ΔMEF: primary MEF from Steel-null embryos. M220: stromal cell line express only membrane-bound Steel factor. ** = p<0.01. (C) PGC number reduced significantly in E8.0 Steel^d/d^ embryos compared to their littermates. “n” indicates the number of embryos used for quantitation. * = p<0.05.

Steel factor controls several aspects of PGC behavior, and both forms of the protein surround them during migration. This suggests the hypothesis that different forms of the protein play different roles. To test this, we used *Steel^d/d^* embryos, in which the membrane-bound form is not present. We bred the *Steel-dickie* mutation into the *Stella-GFP* mouse line in which PGCs express GFP under the control of the *Stella* promoter. We then and counted PGC numbers in bisected E7.5 *Steel^d/d^*, *Stella-GFP* embryos under the confocal microscope. Interestingly, there was no significant decrease of PGC numbers in the E7.5 *Steel^d/d^* embryos (23.3±2.1 per embryo) compared to wild type (26±3.6 per embryo, p = 0.229) and heterozygous littermates (25.4±4.5 per embryo, p = 0.446). This result is shown in **Fig. 2A** and indicates that the soluble Steel factor produced in the *Steel-dickie* mutants is sufficient to maintain PGC numbers immediately after their specification in the allantois. These data should be compared with those from *Steel-null* embryos at the same stage [4], in which the PGC numbers were already reduced by E7.5, and could be rescued by removing an allele of *Bax*, indicating that they were already starting to undergo apoptosis in the absence of Steel factor.

To further confirm that soluble Steel factor is sufficient to maintain PGC number, we cultured isolated PGCs on primary MEFs derived from *Steel-null* embryos (ΔMEF), and added increasing concentrations of soluble recombinant mouse Steel factor into the culture medium. After culture for 24 hours, PGC numbers in Steel factor-treated cultures were significantly higher than those without Steel factor (**Fig. 2B**). There were no obvious differences between different Steel factor concentrations, and the lowest concentration (50 ng/ml) was enough to show the effect (**Fig. 2B**). To test whether membrane-bound Steel factor alone is able to maintain PGC number, we cultured isolated PGCs on M220 cells which express only membrane-bound Steel factor [16], [24]. PGC numbers on M220 cells were similar to those on ΔMEF cells with added Steel factor after 24 hours culture (**Fig. 2B**), and significantly higher than those in the Steel factor-free groups (**Fig. 2B**). These data show that either form of Steel factor is capable of maintaining PGC survival in culture.

*Steel-dickie* mutants are sterile, and the number of PGCs colonizing the genital ridges is dramatically reduced [11], [23]. To investigate when PGC number starts to decrease, we counted PGC numbers in E8.0 embryos. There were 50.5±7.5 PGCs per embryo found in wild type embryos, and this number was significantly reduced to 32±9.0 in *Steel^d/d^* mutants (p = 0.02) (**Fig. 2C**). Given that soluble Steel factor is sufficient to maintain PGC numbers at E7.5, the reduction of PGC number may be caused by a decrease in proliferation. Another possibility is that the PGCs fail to migrate in *Steel^d/d^* embryos, which causes some of them to remain in the allantois when Steel factor is turned off there [4], and thus die by apoptosis, in the same manner which occurs later to PGCs left in the midline [7].

### *Steel-dickie *mutants show defective PGC motility

**Figure 3:**
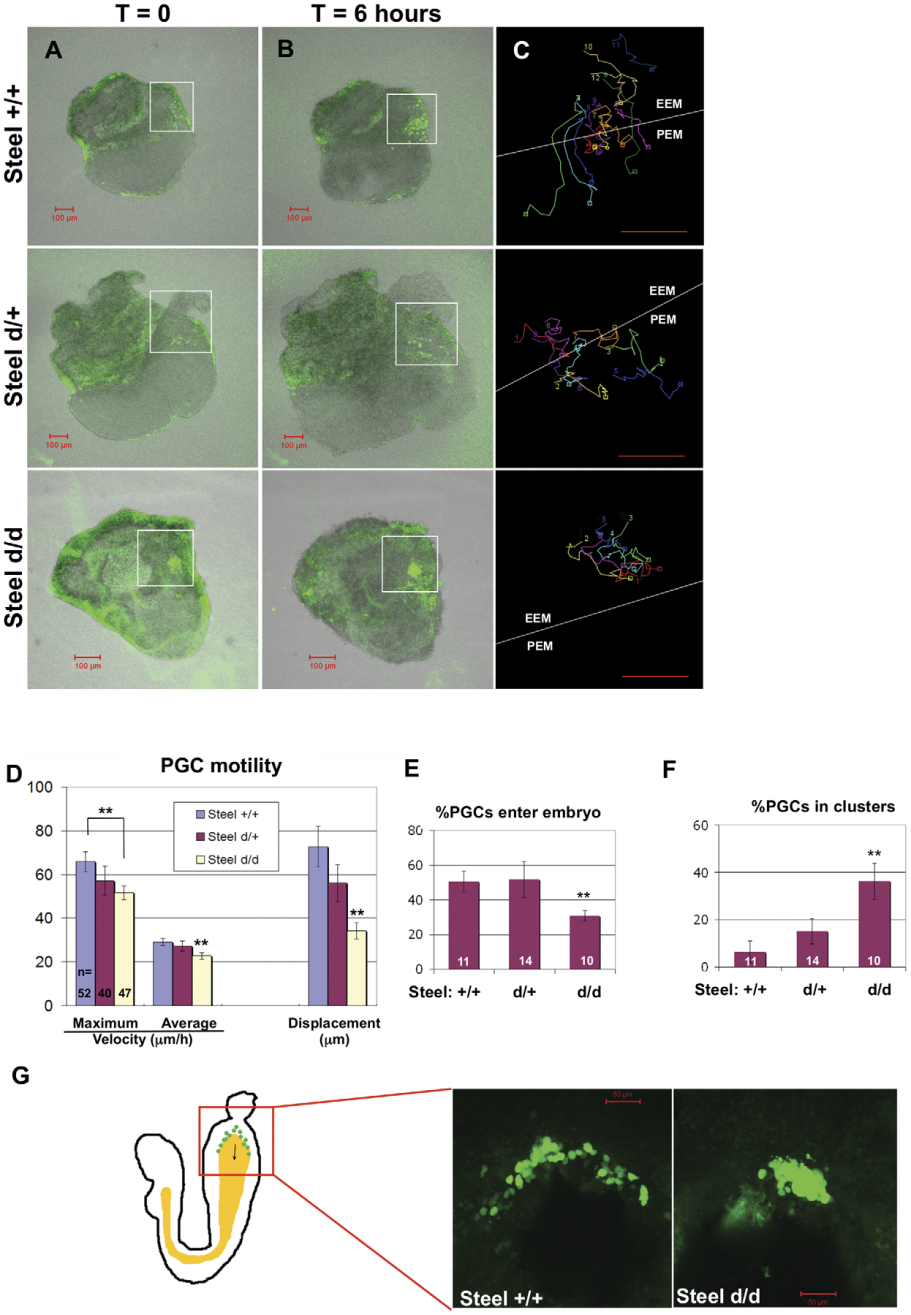
PGC migration in *Steel^d/d^* embryos at E7.5. (Column A, B) Frames at t = 0 and t = 6 hours respectively from movies of E7.5 embryos with different Steel-dickie genotypes. (Column C) Tracks were made from PGCs in the allantois (white boxes) that remained in the plane of the confocal image throughout the movies. The white line in C indicates the boundary between the extraembryonic tissues (EEM), and the posterior end of the embryo (PEM). Scale bars in (A–C): 100 µm. (D) The maximum velocity, average velocity, and displacement of E7.5 PGCs with different Steel-dickie genotypes. PGCs in Steel^d/d^ embryos showed significantly decreased velocities and displacement compared to wild type littermates. “n” indicates the number of PGCs used for quantitation. Units on the “Y” axis vary based upon parameter, and are indicated below the bar charts. ** = p<0.01. (E) The percentage of PGCs which enter the posterior of the embryo is significantly reduced in Steel^d/d^ embryos. (F) The percentage of PGCs which form clusters is dramatically increased in Steel^d/d^ embryos. “n” indicates the number of embryos used for quantitation for (E) and (F). ** = p<0.01. (G) PGCs in E8.0 wild type and Steel^d/d^ embryos. The left diagram shows an E8.0 embryo with PGCs migrating along the hind gut (yellow). Arrow shows the direction of PGC migration. Red box indicates the area shown in the image on the right.

To test whether membrane-bound Steel factor is required for normal PGC migration, time-lapse analyses were carried out using sagittally bisected E7.5 *Stella-GFP^+^, Steel^d/d^* embryos and their littermates. The time-lapse movies are accessible as Video S1, S2, S3, and the examples of movie frames are shown at time = 0 and time = 6.0 hours (Fig. 3A and B). The movements of individual PGCs were manually traced in serial confocal images from each embryo. Tracings were taken only from *PGCs that* remained in focus and could be distinguished from others for the duration of the filming (**Fig. 3C**). The PGCs aggregate together in *Steel^d/d^* embryos; therefore, not all of the PGCs in *Steel^d/d^* embryos were suitable for analysis, and therefore fewer tracks are shown from *Steel^d/d^* embryos in **Fig. 3C**. The velocities and displacements of PGCs were calculated based on these tracings (**Fig. 3D**). Although PGC numbers in *Steel^d/d^* embryos remained similar to those of wild type littermates, the maximum velocity (51.7±3.2 µm/h), the average velocity (22.8±1.5 µm/h) and the displacement (34.3±3.7 µm) of PGCs in E7.5 *Steel^d/d^* embryos were all significantly reduced when compared to wild type (Maxi V = 65.9±4.5 µm/h, *p* = 3.3×10^−6^; Ave V = 29.2±1.2 µm/h, *p* = 2.3×10^−7^; Dis = 72.7±9.2 µm, *p* = 8.5×10^−11^). Since soluble Steel factor was present in these embryos, and PGC numbers did not change, these data show that the membrane-bound form of Steel factor is specifically required for normal PGC motility at the beginning of their migration.

We then scored the numbers of PGCs that migrated across the boundary from the extraembryonic allantoic region (EEM) into the posterior end of the embryo (PEM) in embryos of different genotypes. As a result of reduced PGC motility, the percentage of PGCs that reached the posterior epiblast was significantly decreased in *Steel^d/d^* embryos (30.8±2.9%) when compared with wild type (50.5±6.0%, p = 0.002) and heterozygous littermates (51.7±10.4%, p = 0.009) (**Fig. 3E**). These data demonstrate that decreased motility of the PGCs in the absence of membrane-bound Steel factor leads to a dramatic decrease in the numbers of PGCs reaching the hind gut endoderm.

Unlike PGCs in wild type embryos which were highly motile and moved individually, PGCs in E7.5 *Steel^d/d^* embryos aggregated together into clumps in the proximal region of the allantois (**Fig. 3A, B**) in the same way as previously seen in *Steel^-/-^* embryos [4]. The percentages of PGCs in clusters of more than 3 PGCs were scored in E7.5 embryos of each genotype (**Fig. 3F**). In wild type embryos, around 6.5±4.5% of PGCs form clusters. This number was significantly increased to 36.2±4.7% in the *Steel-dickie* mutants (*p* = 2.2×10^−6^).

We then looked at the PGC locations in E8.0 embryos, when PGCs normally enter into the hind gut and migrate along it anteriorly (as shown in the cartoon in **Fig. 3G**, arrow indicates the direction of migration). In wild type embryos, most PGCs were found localized along the hind gut diverticulum extending anteriorly from its posterior margin towards the mid gut (**Fig. 3G**). In contrast, the majority of PGCs in *Steel^d/d^* embryos were observed in a big cluster at the junction of the posterior margin of the hind gut diverticulum and the allantois, indicating a failure of their anterior migration at this stage (**Fig. 3G**). These data further confirm that membrane-bound Steel factor is required for normal PGC migration *in vivo*.

### Addition of soluble recombinant mouse Steel factor rescues PGC motility defects at E7.5

**Figure 4:**
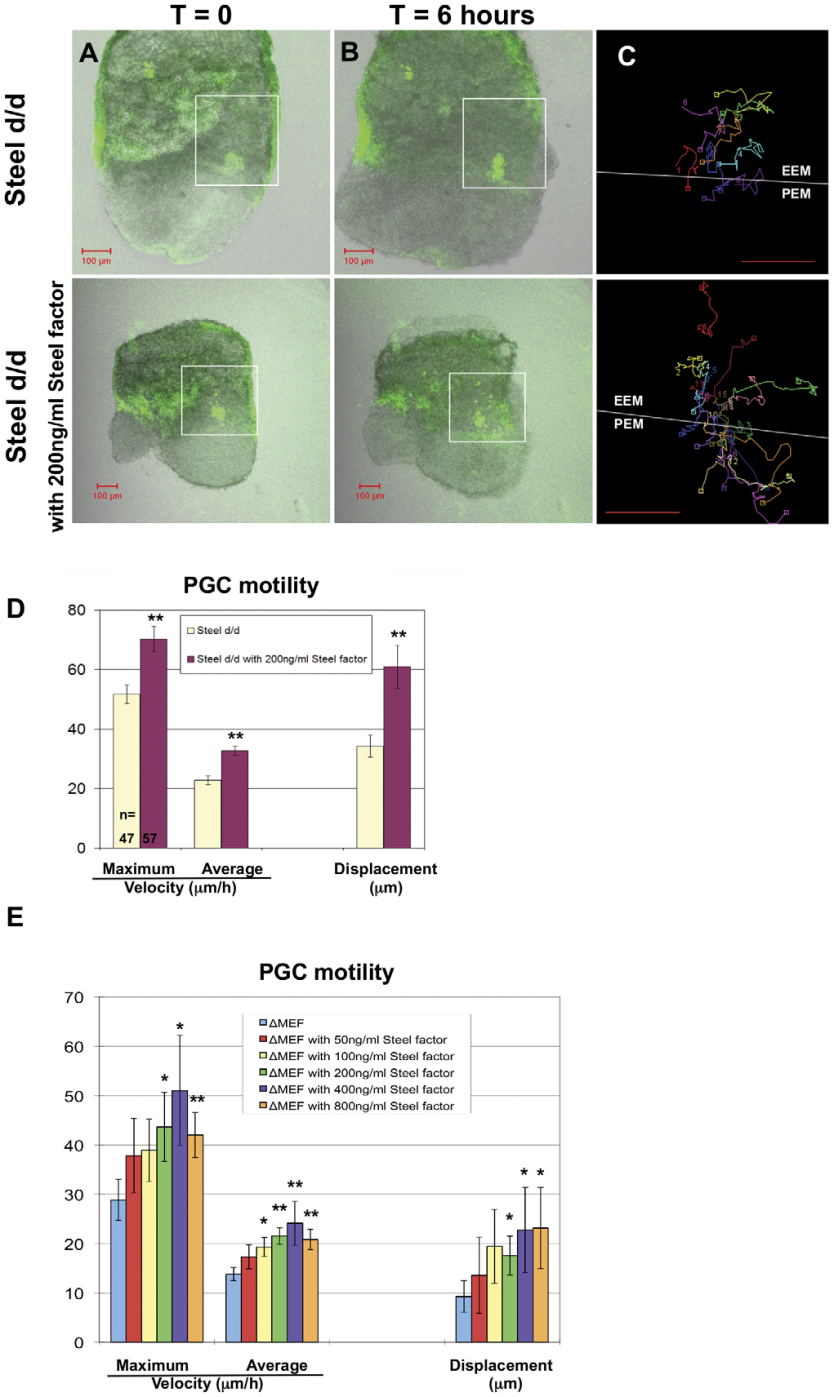
Effects of soluble recombinant Steel factor on PGC motility in *Steel^d/d^* embryos at E7.5 and *in vitro* culture. (Column A, B) Frames at t = 0 and t = 6 hours respectively from movies of E7.5 Steel^d/d^ embryos with or without addition of 200 ng/ml soluble recombinant Steel factor. (Column C) Tracks were made from PGCs in the allantois (white boxes) that remained in the plane of the confocal image throughout the movies. The white line indicates the boundary between the extraembryonic tissues (EEM), and the posterior end of the embryo (PEM). Scale bars in (A–C): 100 µm. (D) The maximum velocity, average velocity, and displacement of PGCs in E7.5 Steel^d/d^ embryos were significantly increased by adding of 200 ng/ml soluble recombinant Steel factor into culture medium for 6 hours. “n” indicates the number of PGCs used for quantitation. Units on the “Y” axis vary based upon parameter, and are indicated below the bar charts. ** = p<0.01. (E) The maximum velocity, average velocity, and displacement of PGCs after 24 hours in vitro culture with increasing concentration of soluble recombinant Steel factor on Steel-null MEFs (ΔMEF). Units on the “Y” axis vary based upon parameter, and are indicated below the bar charts. * = p<0.05.

There are two obvious possible mechanisms whereby membrane-bound Steel factor could be required for PGC motility. First, it may play a specific role on the cell surface of the secreting cell, perhaps mediating the adhesiveness of PGCs to their surrounding cells. Second, it may sustain a higher local ligand concentration, which is essential for normal PGC motility, while the concentration of soluble Steel factor is lower, due to diffusion away from the secreting cells. To distinguish between the two possibilities, we added increasing amounts of soluble recombinant Steel factor to *Steel-dickie* mutant embryos at E7.5. If the Steel factor specifically functions as a membrane component, this should not rescue the motility defects seen in *Steel-dickie* mutant embryos. If the primary function of membrane-bound Steel factor is to maintain a high level of ligand immediately around the PGC, it should rescue the motility defects. The time-lapse movies are accessible as Video S4 and S5, and the examples of movie frames are shown at time = 0 and time = 6.0 hours (**Fig. 4A and B**), and the velocities and displacements of PGC migration were calculated based on the tracks of individual PGC movement (**Fig. 4C**).

Addition of 200 ng/ml Steel factor significantly increased the motility of PGCs in E7.5 *Steel^d/d^* embryos (**Fig. 4D**). The maximum velocity was increased from 51.7±3.2 µm/h to 70.2±4.3 µm/h (*p* = 2.4×10^−9^), the average velocity was increased from 22.8±1.5 µm/h to 32.7±1.5 µm/h (*p* = 6.0×10^−15^), and the displacement of PGC movement was increased from 34.3±3.7 µm to 60.9±7.2 µm (*p* = 2.2×10^−8^). These findings suggest that the primary role of membrane-bound Steel factor is to maintain an optimal local ligand concentration for PGC migration, and that Steel factor activates two different functions in PGCs at different concentrations.

To further confirm that higher concentrations of Steel factor activate PGC motility, we cultured PGCs *in vitro* on *Steel-null* MEFs with the addition of increasing concentrations of soluble Steel factor. In contrast to the data in **Fig. 2B**, which show that 50 ng/ml soluble Steel factor was sufficient for maintaining PGC number (there were no differences in the survival effects between different concentrations of Steel factor), higher doses of added Steel factor (≥200 ng/ml) were required to show a significant increase of both PGC velocity and displacement compared to those without Steel factor (**Fig. 4E**, p<0.05) (There were no significant differences in the motility effects between the concentrations higher than 200 ng/ml). Together, these data suggest that Steel factor controls different aspects of germ cell behavior at different concentrations. Low concentrations are sufficient to maintain PGC survival, while higher concentrations provided by the membrane-bound form activate both survival and motility.

### Global addition of soluble recombinant mouse Steel factor caused distal PGCs to migrate randomly at E7.5

**Figure 5:**
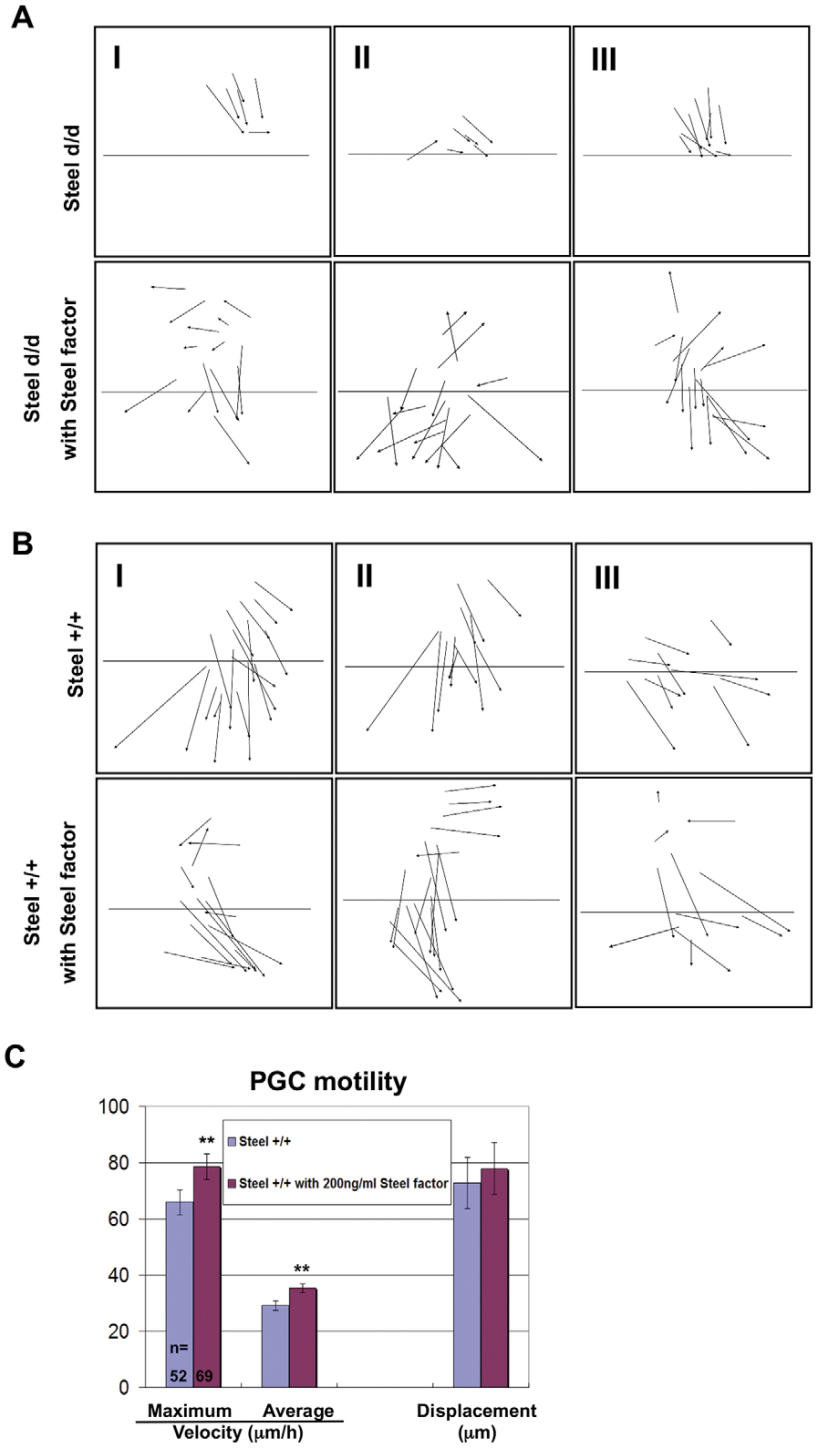
Effects of soluble recombinant Steel factor on PGC directions. Directions of individual PGC migration in Steel^d/d^ embryos (A) or wild type embryos (B) with or without addition of 200 ng/ml soluble recombinant Steel factor. The boundary between the extraembryonic tissues (EEM), and the posterior end of the embryo (PEM), is marked by a line. Column I, II, and III are representative images from 3 different embryos of the same genotype labeled on the left. (C) The maximum velocity, average velocity, and displacement of PGCs in E7.5 wild type embryos with or without addition of 200 ng/ml soluble recombinant Steel factor into culture medium for 6 hours. “n” indicates the number of PGCs used for quantitation. Units on the “Y” axis vary based upon parameter, and are indicated below the bar charts. ** = p<0.01.

The addition of soluble Steel factor to *Steel^d/d^* embryos in culture dramatically increased PGC motility (**Fig. 4**). An interesting feature of this was revealed by indicating the overall direction of their movements by drawing an arrow to link the start point and the end point of individual PGC movement tracks. The results are shown in **Fig. 5A** (Column I, II, and III are representative images from 3 different embryos). Without Steel factor addition, most of PGCs migrated more slowly than in wild type embryos, but in the correct direction; proximally towards the posterior region of the embryo. However, in *Steel^d/d^* embryos with added soluble Steel factor, some PGCs located in distal portion of the allantois moved in the wrong directions, while PGCs closer to posterior embryo migrated correctly (**Fig. 5A**). The most likely explanation for this is that distally-located PGCs are out of range of the directionality cues that guide PGCs into the posterior endoderm at this stage. These PGCs would normally die by apoptosis as Steel factor is turned off, but are maintained by the addition of exogenous Steel factor. The alternative explanation, that membrane-bound Steel factor itself provides guidance information seems less likely, since the germ cells closest to the embryo do continue to migrate in the right direction.

To investigate whether a globally higher concentration of Steel factor has the same effect in a wild type background, we added 200 ng/ml soluble recombinant Steel factor into E7.5 wild type embryos and analyzed the directions of PGC migration. PGCs in wild type embryos with added Steel factor also showed randomized migration only when they were located in the most distal region of the allantois, while PGCs without Steel factor addition predominantly migrate towards the correct target (**Fig. 5B**). We also traced individual PGC movements in wild type embryos after addition of soluble Steel factor. The maximum velocity and average velocity of PGC motility were accelerated with Steel factor addition (*p* = 1.7×10^−4^, 1.1×10^−7^, respectively) (**Fig. 5C**), suggesting that PGC motility responds to Steel factor in a dose-dependent manner. There was no significant increase in the displacement of PGC migration with Steel factor addition (*p* = 0.44).

### Global addition of soluble recombinant mouse Steel factor caused PGCs in the genital ridges to reacquire motility

**Figure 6:**
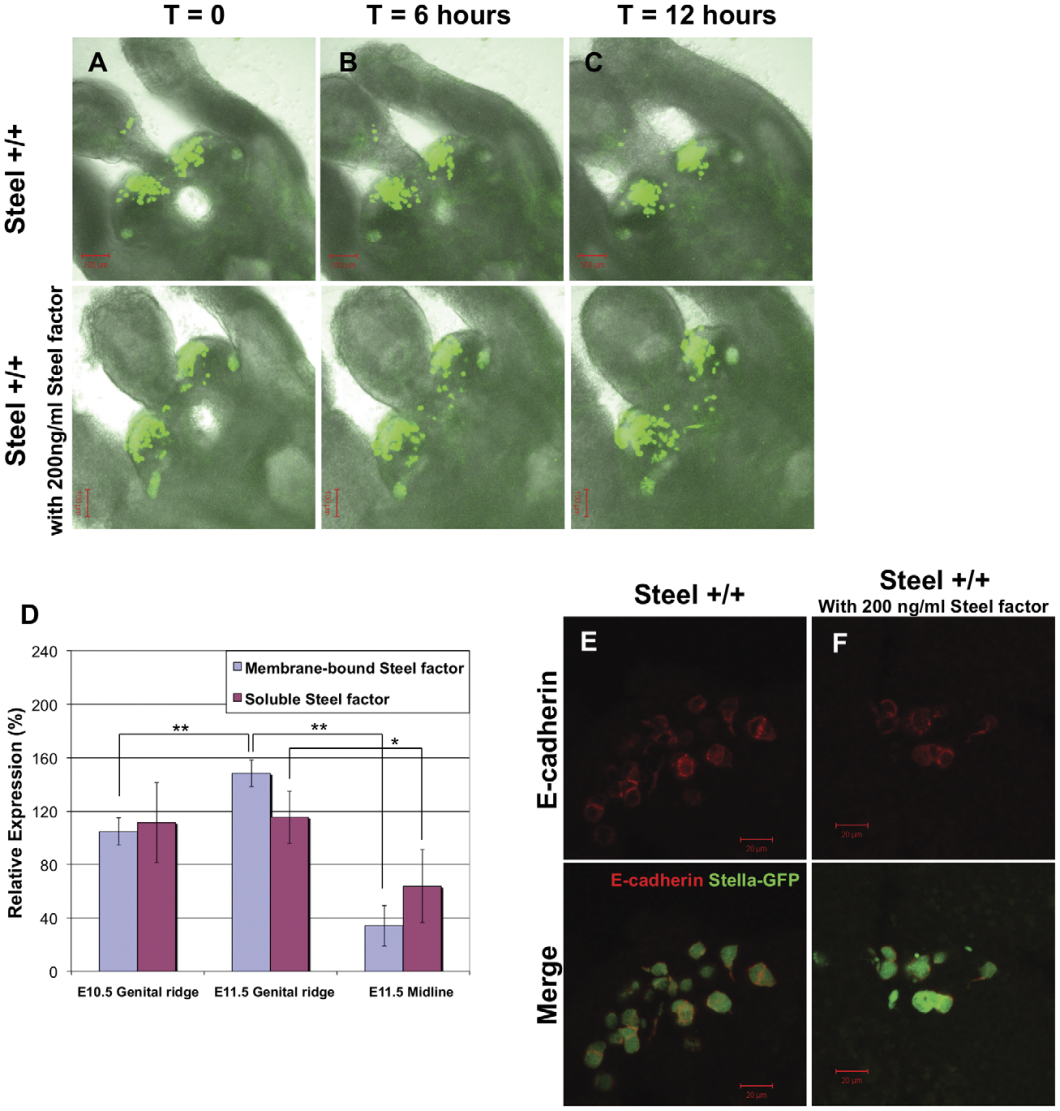
Effects of soluble recombinant Steel factor on PGC motility in wild type embryos at E11.0. (Column A-C) Frames at t = 0, t = 6 and t = 12 hours respectively from movies of E11.0 wild type embryo slices with or without addition of 200 ng/ml soluble recombinant Steel factor. (D) The relative expression level of membrane-bound and soluble Steel factor mRNA in E10.5 genital ridges, E11.5 genital ridges and E11.5 midline mysenchyme as determined by real-time RT-PCR. *p<0.05, **p<0.01. (E and F) E-cadherin expression in PGCs without (E) or with (F) addition of Steel factor. Upper panels show E-cadherin (red) staining. The lower panels show the merged images with PGC marker Stella-GFP (green). No difference in expression of E-cadherin was observed between groups.

PGCs aggregate together and stop migrating when they reach the genital ridges around E11.0 [5]. The requirement for Steel factor for motility raised the hypothesis that the cessation of PGC migration could be due to down-regulation of Steel/c-kit signaling. To test this hypothesis, we added 200 ng/ml soluble recombinant Steel factor into E11.0 wild type embryo slices and made time-lapse movies. The movies are available as Video S6 and S7, and the examples of movie frames are shown at time = 0, 6.0, and 12.0 hours (**Fig. 3G A–C**). Without addition of Steel factor, PGCs moved slowly and gradually coalesced with each other to form big clusters in the genital ridges within this time period. In slices with global addition of soluble Steel factor, there was an obvious acceleration of the motility in some of the PGCs, which migrated out of the genital ridges into surrounding tissues. This data suggest that the normal cessation of PGC migration, and their aggregation, may be caused by regulation of Steel factor signaling. One obvious possibility is that membrane-bound Steel factor expression is down regulated in the genital ridges. To test this, we dissected the genital ridges from E10.5 embryos, and both genital ridges and dorsal midline regions from E11.5 embryos. qPCR was used to assay the expression levels of both forms (**Fig. 6D**). Consistent with previously published data, the expressions of both forms of Steel mRNA were significantly reduced in the E11.5 midline compared to E11.5 genital ridges (*p*<0.05) [7]. However, no decrease in either form of Steel mRNA was observed in E11.5 genital ridges compared to E10.5 genital ridges. Instead, the membrane-bound mRNA was significantly increased (*p*<0.01). This suggests that the cessation of PGC migration is not due to reduced expression of membrane-bound Steel factor in the genital ridges at E11.5.

E-cadherin is expressed by migrating PGCs, and blockade of its function by the ECCD-1 blocking antibody is known to cause a similar spreading of the PGCs into adjacent tissues at E11.5 [25]. To examine whether the addition of Steel factor promotes PGC motility through down-regulating E-cadherin expression, we applied antibody staining for E-cadherin on embryo slices after treatment with soluble Steel factor. As shown in **Fig. 6E and F**, there is no significant change of E-cadherin expression in PGCs between the groups, suggesting that the maintenance of motility, rather than aggregation by Steel factor is not by control of E-cadherin expression.

## Discussion

Many studies in the past have reported that Steel factor plays an essential role in PGC development [7], [11], [12], [14]-[16]. Most recently, work from our lab revealed that the Steel factor-expressing cells establish a “traveling niche” surrounding migratory PGCs which provides a short-range signal to regulate PGC survival, proliferation and motility throughout their migration [4]. However, how these different behaviors are regulated by the same ligand, and how Steel factor functions as a short-range signal remain uncertain. In other cell populations, the two forms of Steel factor, membrane-bound and soluble form, have been shown to have different functions [17], [19]–[22], although the mechanisms whereby they do so have remained unclear. This suggested that different aspects of PGC behavior might also be regulated through different forms of Steel factor. The *Steel-dickie* mutation, which only produces soluble Steel factor due to a deletion in cytoplasmic and transmembrane domain of *Steel* allele, offers a great tool to address this question. Previous work has shown that transcription from the *Steel-dickie* allele is at the same level as wild type. Here we showed, by western blotting using supernatant fractions of E12.5 whole embryos, that Steel factor protein is produced in the mutant embryos, and that there is an increased amount of the soluble form, compared to the wild type embryo. This is the expected result if the mutation generates only the soluble form, the translation levels are similar, and if protein stability is not altered. In this case, all the translated protein will be in the soluble form in the *Steel-dickie* mutant embryo. To test the functions of the two forms, we analyzed PGC number and motility in E7.5 Steel^d/d^ embryos. The results showed that the soluble form of Steel factor present in *Steel^d/d^* embryos maintained the survival but not the motility of PGCs. This shows that Steel factor does not need to be membrane-bound for PGC survival, but that the membrane-bound form is essential for their motility.

The results presented here also offer a mechanistic explanation of the somewhat puzzling effects of the *Steel-dickie* mutation. It has long been known that this mutant is viable but sterile, implying that membrane-bound Steel factor plays different roles in the PGCs and hematopoietic cell lineages. However, Steel factor is known to be a survival factor in both cell types [14], [15], [26]–[28], and the soluble Steel factor protein present in *Steel^d/d^* embryos is clearly sufficient to maintain their survival (shown in this work). We propose that the difference is due to the fact that membrane-bound Steel factor provides a niche for maintaining active motility, and in its absence, the PGCs lose motility, and do not move to the next location in the pathway. We have shown previously that Steel factor maintains PGC survival only at short range [7], and that expression of Steel factor moves along the PGC migratory pathway [4]. With reduced motility, PGCs in *Steel^d/d^* embryos will not keep up with the wave of Steel factor expression, and will die because this essential survival factor is turned off before they have moved to the next site of expression. All the observations to date support this hypothesis. First, in E7.5 *Steel^d/d^* embryos, no obvious reduction of PGC number were observed, implying that the soluble Steel factor produced in the *Steel-dickie* mutants is adequate to maintain PGC survival at this stage. Although there is no significant decrease in cell number, PGCs in *Steel^d/d^* embryos show remarkably reduced motility and instead form a large clump of non-motile cells. Second, *Steel^d/d^* PGCs remain clustered at the boundary between allantois and hind gut at E8.0, while most PGCs in wild type embryos have already migrated anteriorly along the hind gut endoderm. Third, many PGCs are found in ectopic locations with apoptotic morphology in *Steel^d/d^* embryos [11].

The finding that addition of soluble Steel factor to E7.5 *Steel^d/d^* embryos is capable of restoring PGC motility is interesting, because it suggests the membrane-bound Steel factor is essential to maintain an optimal local concentration in a defined region, rather than play a distinct role to regulate PGC motility. The addition of large amounts of soluble recombinant Steel factor can restore PGC motility in *Steel^d/d^* embryos, showing that the local ligand concentration is crucial for Steel factor regulation. In support of this view, a previous study showed that *in vivo* administration of soluble recombinant Steel factor into *Steel^d/-^* mice caused a notable reduction of the severity of their macrocytic anemia and a great increase of mast cell number at the injection sites [29]. The high levels of Steel factor required for PGC motility is normally maintained not by increased synthesis, but by maintenance of a high local concentration by presenting it on the membranes of adjacent cells in a restricted area, thus defining the region in which PGCs can maintain their motility. Movement of this “motility niche” would thus create the pathway along which PGCs are able to move.

When treating the E7.5 *Steel^d/d^* and wild type embryos with added soluble Steel factor, some PGCs in the most distal region of allantois tended to migrate randomly, instead of moving proximally towards the posterior epiblast. It may be that these PGCs are already too far away from the normal chemotactic signals, which attract PGCs to the posterior epiblast. Without the added soluble Steel factor, these cells would normally die by apoptosis because they are in an ectopic location where Steel factor expression is being turned off. However, the globally added soluble Steel factor allowed them to survive and migrate randomly. These findings accentuate the importance of maintaining a high local concentration only in a defined region to make sure that PGCs which receive the chemotactic signals maintain motile, whereas the ones out of the range of direction cues cease motility and die.

One observation that remains unexplained is the formation of large clumps of PGCs in the allantois in both *Steel^-/-^* and *Steel^d/d^* embryos. We have previously shown that PGCs activate expression of E-cadherin when they leave the hind gut. There is a failure of the normal aggregation of germ cells as they enter the genital ridges when E-cadherin function is blocked [25]. In a previous study of *Steel-null* mutant embryos, we stained for E-cadherin protein in PGCs of different genotypes to check whether PGC clumping at E7.5 was due to an upregulation of E-cadherin in the absence of Steel factor. However, no significant change in E-cadherin expression level was observed [4]. To further confirm that E-cadherin is not responsible for the increased cluster formation and decreased motility, we treated wild type E7.5 embryos with Ack2, the inhibitor of c-kit receptor, and ECCD1, an antibody which blocks the function of E-cadherin, to test whether loss of E-cadherin function could rescue the defects caused by loss of Steel factor signaling. Compared to embryos which were only treated with Ack2, the dual treatment group did not show any significant change in motility, displacement and cluster formation (data not shown), indicating the defects shown in *Steel^-/-^* and *Steel^d/d^* embryos are not due to a premature up-regulation of E-cadherin function. The mechanism of PGC cluster formation in *Steel-null* and *Steel-dickie* mutants needs to be investigated in future studies.

It is important to maintain PGC motility during their migration. It is also crucial to ensure PGCs stop migration when they coalesce in the genital ridges. Interestingly, addition of soluble Steel factor into embryo slices at E11.0 resulted in reacquisition of motility in PGCs, indicating that PGCs can still respond to high levels of Steel factor, and that modulation of Steel-ckit signaling may control the ending of migration. However, there is no decrease in the expression of Steel factor mRNA in E11.5 genital ridges as determined by real-time RT-PCR, suggesting that the ending of PGC migration is not due to down-regulation of Steel factor expression at transcriptional level. There are a number of possible explanations for this paradoxical finding. First, PGCs could still be motile in the E11.5 genital ridge, but are constrained by other factors. Second, there may be less membrane-bound Steel factor at the protein level. Third, an inhibitor of Steel factor signaling could be present in the genital ridges, which acts independently of Steel factor expression level. Fourth, when PGCs arrive in the genital ridges, the amount of membrane-bound Steel factor available per PGC may decrease. As PGCs adhere to each other, they will be less likely to be adjacent to a membrane-bound Steel factor-expressing somatic cell. Whatever the mechanism of clumping, it is overcome by addition of increased soluble Steel factor, so is likely to include the inhibition of Steel factor signaling. E-cadherin function has been shown to be essential for PGC coalescence in the genital ridges [25]. Although there is no obvious change in E-cadherin expression in PGCs with Steel factor addition, we cannot exclude the possibility that Steel factor promotes PGCs to migrate out of the cluster by interrupting E-cadherin function without affecting its membrane expression. Further studies need to be performed to address these questions.

These studies reveal two important aspects of the “traveling niche”. First, the Steel factor activates two different functions in PGCs at different concentrations, and second that the moving expression of membrane-bound Steel factor maintains PGC motility in a defined region of the embryo. PGCs escaping from this region, or that get left behind on the migratory route, cease motility, and are removed by apoptosis as the expression wave moves on towards the genital ridges. Thus the colonization of the genital ridges is as much due to the control of survival and motility as it is to chemotactic signals.

## Materials and Methods

### Ethics Statement

All experiments were carried out in strict accordance with institutional guidelines under Institutional Animal Care and Use Committee (IACUC) approval at Cincinnati Children's Hospital Research Foundation (CCHRF). IACUC at CCHRF approved the study described in this manuscript with Animal Use Protocol number 9A02019.

### Mouse breeding, embryo preparation and genotyping

*Stella-GFP* transgenic mice [30] on a B6/CBA background were crossed with *Steel-dickie* (*Kitl^Sl-d^* or *Steel^d/d^*) heterozygotes (Jackson Laboratories, Stock number 000160) on a C57BL/6J background to obtain mice that were Stella-GFP^+^, *Steel^d/+^*. These mice were interbred to yield *Stella-GFP^+^*, *Steel^d/d^* embryos. Embryonic day 0.5 (E0.5) was assumed to be noon of the morning a vaginal plug was observed. Genomic DNA was isolated from tail snips (adults), embryo halves (E7.5 embryos) and heads (E8.0 and later stages), and genotypes were determined by PCR. Genotyping primers used were as follows: Stella-GFP: F-5′TGCATCGGTAACCCACAGTA-3′, R-5′ GAACTTCAGGGTCAGCTTGC 3′; Steel-Dickie: WT-F-5′TGCAAAGGCTGCACAGTAAG-3′, DEL-F-5′GGTGTGAGTGAGCCAAGAGC-3′, Common-R-5′CCAAGCCTTTCTCCAGTCAT-3′. Stella-GFP expression was determined by the presence of a 289 bp fragment. For Steel-dickie, wild type and deleted alleles were identified by 180 and 770 bp fragments, respectively.

### Embryo and slice culture

Embryo culture was used as described previously [4]. E7.5 embryos were cut sagittally into halves using a scalpel. One half of each embryo was used for culture and the other half was used for genotyping. The embryo halves were put onto millicell culture inserts pre-coated with collagen IV (BD), and the inserts were then placed into a metal stage which contains glass-bottom chambers with 600 µl culture medium (Hepes-buffered DMEM/F-12 (Gibco) medium with 0.04% lipid-free BSA and 100 U/ml penicillin/streptomycin (Gibco)). For the Steel factor addition assay, 200 ng/ml soluble mouse recombinant Steel factor (R&D Systems) was added into the embryo culture medium immediately before time-lapse analysis was started. Embryo halves were maintained in a humidity-controlled environment, and at 37°C throughout time-lapse analysis. For E11.0 embryos, embryo slices were prepared as described previously [7], and cultured under the same condition as embryo culture.

### Time-lapse analysis of migrating PGCs

Time-lapse analyses were carried out using a Zeiss LSM510 confocal system attached to a Zeiss axiovert microscope. Embryo slice images were acquired every 5 minutes for 6 hours, and movies were analyzed using NIH Image J as described [4], [5]. Experiments were repeated at least three times, and three to eight embryos were analyzed per group.

### Isolation of PGCs

Embryos from *Stella-GFP* mice were harvested between E9.5 to E10.5, and tissues containing PGCs were dissected in PBS, washed 3 times in PBS, and trypsinized by adding a small volume of 0.25% trypsin-EDTA (Gibco) and incubated at 37°C for 5 minutes. Tissues were then pipetted in 200 µl PBS containing 1% Fetal Bovine Serum (FBS) (Gibco) for about 50 times using a 200 µl tip to make single cell suspension. The single cell suspension was then passed through a cell trainer cap into a 12×75 mm Falcon test tube (Falcon) and analyzed by Flow cytometry. PGCs were isolated based on their GFP expression into PGC culture medium (phenol red-free L-15 medium (Gibco) with 20% knockout serum replacement (Gibco), 0.1 mM non-essential amino acids (Gibco), 0.1 mM 2-mercaptoethanol (Gibco), and 100 U/ml penicillin/streptomycin (Gibco)).

### In vitro culture of PGCs

Primary mouse embryonic fibroblast cells and M220 cells (kindly provided by Dr. David A. Williams) were routinely grown in DMEM with 10% FBS (Gibco), 100 U/ml penicillin/streptomycin (Gibco), and 2 mM L-glutamine (Gibco). Confluent cell monolayers were inhibited from proliferation by treatment with 5 µg/ml mitomycin C (Sigma) for 2 hours at 37°C. Cells were then washed with PBS, harvested by trypsinization, and adjusted to a concentration of 2×10^5^ cells/ml, and 300 µl per chamber were plated into LabTek II 8 chamber coverglass (Nunc). Flow-sorted PGCs were added onto the treated feeder cell layers 24 hours after plating and cultured in PGC culture medium. PGC number was counted under fluorescent microscopy, and PGC motility was analyzed by time-lapse movies.

### Western Blots

To detect levels of Steel factor protein, embryos were harvested at E12.5 and each embryo was homogenized with 50 µl lysis buffer (1% Triton X-100, 1 mM PMSF and 1∶100 dilution of protease inhibitor cocktail (PIC, Sigma) in ice-cold PBS). Lysates were cleared by centrifugation at 12,000 rpm for 5 minutes at 4°C. The supernatants were then transferred into clean 1.5 ml microfuge tubes. 10 µl of 4x sample buffer was added to 20 µl supernatats, mixed and boiled for 5 minutes. The samples were then loaded on 12% SDS PAGE gels and separated for 2 hours at 90 volts. Gels were blotted onto nitrocellulose membranes and all the membranes were blocked with 2% BSA in PBS with 0.1% Tween 20 for 2 hours at room temperature. For Steel factor detection, membranes were incubated with goat anti-mouse Steel factor antibody (R&D Systems) at a dilution of 1∶1000 in blocking buffer overnight at 4°C. Donkey anti-goat HRP secondary antibody was used at a dilution of 1∶5000. Signal detection was carried out using ECL developing solution (Amersham). As a loading control, membranes were stripped after signal detection and incubated with anti-α-tubulin antibody at 1∶10,000.

### RT-PCR and real-time RT-PCR

For RT-PCR analysis, allantoides from E7.5 embryos, hind guts from E8.5 embryos, hind guts and dorsal body walls from E9.5 embryos, and genital ridges from E10.5 embryos were dissected. For real-time RT-PCR, genital ridges from E10.5 and E11.5 embryos, and midline region of dorsal body walls from E11.5 embryos were dissected. RNA was extracted from the dissected tissues using RNeasy Kit (Qiagen) and reverse-transcribed using Superscript III First-Strand Synthesis Systems (Invitrogen). Regular PCR reactions were performed using Redmix Plus (GeneChoice). Real-time RT-PCR was performed using a LightCycler (Roche). Water-blank and RT-minus controls were included in all runs. All real-time RT-PCR results are presented as percentage compared with the level in E10.5 genital ridges after normalization to the expression of glyceraldehyde 3-phosphate dehydrogenase (GADPH). Primers used were as follows:

Membrane-bound Steel factor: F-5′TCCCGAGAAAGGGAAAGC-3′, R-5′ CTGCCCTTGTAAGACTTGACTG-3′ (predicted fragment length: 149 bp)

Soluble Steel factor: F-5′TTATGTTACCCCCTGTTGCAG-3′, R-5′ CTGCCCTTGTAAGACTTGACTG-3′ (predicted fragment length: 195 bp)

GAPDH: F-5′ACCACAGTCCATGCCATCAC-3′, R-5′TCCACCACCCTGTTGCTGTA-3′ (predicted fragment length: 452 bp)

### Statistical analysis

All results are expressed as mean values ± SEM. Statistical significance was determined with two-tailed paired Student's *t*-test and a probability of *p*<0.05 was considered to be statistically significant.

## Supporting Information

Video S1 **PGC migration in E7.5 wild type embryo.** Time-lapse movie of an E7.5 *Stella-GFP/Steel^+/+^* embryo bisected sagittally, and cultured as described in the Materials and methods. PGCs are green.
(MOV)

Video S2 **PGC migration in E7.5** ***Steel^d/+^*** **embryo.** Time-lapse movie of an E7.5 *Stella-GFP*/*Steel^d/+^* embryo bisected sagittally, and cultured as described in the Materials and methods. PGCs are green.
(MOV)

Video S3 **PGC migration in E7.5** ***Steel^d/d^*** **embryo.** Time-lapse movie of an E7.5 *Stella-GFP*/*Steel^d/d^* embryo bisected sagittally, and cultured as described in the Materials and methods. PGCs are green.
(MOV)

Video S4 **PGC migration in E7.5** ***Steel^d/d^*** **embryo without addition of soluble Steel factor.** Time-lapse movie of an E7.5 *Stella-GFP*/*Steel^d/d^* embryo bisected sagittally, and cultured without addition of soluble Steel factor. PGCs are green.
(MOV)

Video S5 **PGC migration in E7.5** ***Steel^d/d^*** **embryo with 200 ng/ml soluble Steel factor.** Time-lapse movie of an E7.5 *Stella-GFP*/*Steel^d/d^* embryo bisected sagittally, and cultured in the presence of 200 ng/ml soluble recombinant Steel factor. PGCs are green.
(MOV)

Video S6 **PGC migration in E11.0 wild type embryo without addition of soluble Steel factor.** Time-lapse movie of an embryo slice from E11.0 *Stella-GFP*/*Steel^+/+^* embryo dissected, and cultured without addition of soluble Steel factor. PGCs are green.
(MOV)

Video S7 **PGC migration in E11.0 wild type embryo with 200 ng/ml soluble Steel factor.** Time-lapse movie of an embryo slice from E11.0 *Stella-GFP*/*Steel^+/+^* embryo dissected, and cultured in the presence of 200 ng/ml soluble recombinant Steel factor. PGCs are green.
(MOV)
